# Cough Characteristics and Healthcare Journeys of Chronic Cough Patients in Community-Based Populations in South Korea and Taiwan

**DOI:** 10.1007/s00408-022-00586-3

**Published:** 2022-11-03

**Authors:** Woo-Jung Song, Chong-Jen Yu, Suk Hyun Kang

**Affiliations:** 1 Department of Allergy and Clinical Immunology, Asan Medical Center, University of Ulsan College of Medicine, Seoul, Korea; 2 Department of Internal Medicine, and Graduate Institute of Clinical Medicine, National Taiwan University Hospital, Hsin-Chu Branch and National Taiwan University College of Medicine, Taipei, Taiwan; 3 Market Access, MSD, Seoul, Korea

**Keywords:** Chronic cough, Treatment journey, Disease burden, Cough-specific health-related quality of life

## Abstract

**Purpose:**

This study aimed to understand the cough characteristics and health journeys among community-based chronic cough (CC) patients, and their characteristics associated with healthcare visits.

**Methods:**

A population-based cross-sectional study was conducted in 2020, using the South Korea and Taiwan National Health and Wellness Survey (NHWS) and CC surveys. Patients with current CC were defined by daily coughing for > 8 weeks in the past 12 months and currently coughing at the time of survey. The survey items pertained to CC patients’ treatment journey and cough characteristics.

**Results:**

Patients with current CC in South Korea and Taiwan, respectively, had cough duration for 3.45 ± 5.13 years and 5.75 ± 7.28 years and cough severity visual analogue scale (VAS) scores of 4.50 ± 2.15 and 4.46 ± 1.92 out of 0–10 scale, with 70.3% and 57.9% having spoken with a physician about cough. Compared to CC patients who had not visited healthcare professionals for cough, those who visited reported more severe cough (VAS: 3.89 ± 1.71 vs. 4.6 ± 2.02; *p* = 0.009), worse cough-specific quality of life (Leicester Cough Questionnaire: 16.20 ± 3.23 vs.13.45 ± 2.68, *p* < 0.001), greater symptom severity (Hull Airway Reflux Questionnaire: 16.73 ± 15.16 vs. 24.57 ± 13.38; *p* < 0.001), and more urinary incontinence (13.6 vs. 26.5%, *p* = 0.027). More than 50% of patients perceived cough medication(s) as not or a little useful and 25% felt their physicians did not well understand how CC impacts their life.

**Conclusion:**

Cough is frequently severe and persistent among community-based CC patients. They experience several issues in their health journey, including treatment ineffectiveness and physician’s understanding. Further efforts are warranted to reduce CC burden in the community.

**Supplementary Information:**

The online version contains supplementary material available at 10.1007/s00408-022-00586-3.

## Introduction

Cough is a vital protective reflex to prevent aspiration and enhance airway clearance [1, 2]. However, cough is also one of the most common symptoms for patients seeking care [3]. Particularly, chronic cough (CC), defined by cough persisting for more than 8 weeks, is a major cause of morbidity affecting quality of life (QoL) [4]. The prevalence of CC has been estimated to be about 10% globally [5] and 2–5% in East Asia [6].

During the last decades, several international and national guidelines have been developed for guiding the management of CC patients [7–9]. Several studies reported the characteristics of CC among patients visiting clinics [10–14]; however, there is limited knowledge about cough characteristics and healthcare journey in the community. Large general population-based studies [15–17] reported the prevalence of CC and its impact on health-related QoL; they mostly reported the simple presence of CC but lacked detailed cough information because they were designed to investigate general health issues. Further understanding of cough characteristics and health journey experiences of CC patients in the community will help us to understand the disease burden and identify unmet clinical needs.

Herein, the present study sought to understand (i) cough characteristics in CC patients in the community of South Korea and Taiwan, e.g., cough duration, severity, or cough-specific QoL; (ii) their healthcare journeys; and to explore (iii) cough characteristics associated with healthcare professional (HCP) visits.

## Methods

### Study Design

This is a cross-sectional study, including data from the 2020 South Korea and Taiwan National Health and Wellness Survey (NHWS) as well as CC surveys. The NHWS were conducted in a total of 11 countries/territories, including South Korea and Taiwan, collecting self-reported patient characteristics, disease status, and patient-reported outcomes (PROs). The 2020 South Korea and Taiwan NHWS survey was conducted in January–February 2020 and was described elsewhere [18].

To further understand CC patients’ experience including the impact of CC on QoL and healthcare journey, the add-on CC survey was conducted in March–April 2020 to all eligible respondents from the NHWS who met the eligibility criteria. Both the NHWS survey and the CC survey were approved by the Pearl Pathways Institutional Review Board (IN, USA). All respondents completed the NHWS and the CC survey in local language(s)—Korean (South Korea) and traditional Chinese (Taiwan). All respondents provided informed consent prior to participating.

### Study Population

#### NHWS

Potential respondents to the NHWS, aged 18 years or older, were recruited through an existing, general purpose (i.e., not healthcare specific) web-based consumer panel. All panelists explicitly agreed to be a panel member. While recruiting the respondents, a stratified random sampling procedure, with strata by sex and age, was implemented to ensure that the demographic composition is representative of the respective general adult population in South Korea and Taiwan. There were no exclusion criteria.

#### CC Survey

Among the respondents of the 2020 South Korea and Taiwan NHWS, those who self-reported coughing daily for > 8 weeks in the past 12 months and had current cough at the time of surveys (defined as current CC patients) were invited to participate in the add-on CC survey. Respondents who self-reported any form of lung cancer, having interstitial lung disease, or currently taking an ACE inhibitor were excluded. The patients were divided into subgroups based on their cough severity visual analogue scale (VAS) 2 weeks’ prior to the time of survey (mild CC ≤ 4, vs. severe > 4 of 10) [19].

### Cough Parameters and Patient-Reported Outcomes

Baseline demographic and general health-related parameters were collected through the NHWS (please see Methods in Supplementary Information for more details). In the CC survey, cough-specific parameters were collected, such as years experiencing CC, cough severity, impact, cough-related conditions or behaviors, and health journey experiences. The cough severity was assessed by a VAS ranging from 0 (no cough) to 10 (extremely severe cough). Conditions or behaviors related to CC were defined by patients’ responses to the question “Has a doctor ever told you that any of the following conditions or behaviors are related to your chronic cough?”

Cough-specific QoL was measured by Leicester Cough Questionnaire (LCQ), with the total score range of 3–21 points [20]. Symptom severity associated with cough hypersensitivity was measured by Hull Airway Reflux Questionnaire (HARQ) [21]. The HARQ is a 14-item, self-administered instrument that measures specific symptoms related to cough, with the total score ranging from 0 to 70 (please see Methods in Supplementary Information for more details).

The patient journey included HCPs seen and currently seeing for CC, experience with HCPs, diagnostic tests, medications and diagnoses, and patients’ satisfaction with previous treatments.

### Statistical Analysis

Descriptive statistics were reported using counts and percentages for categorical variables and means and standard deviations (SDs) (or standard errors [SEs] as indicated) for continuous variables. Patient experience, health journey, and medications were also summarized descriptively. Multivariate comparisons of LCQ and HARQ scores were conducted between mild CC (VAS ≤ 4) and severe CC (VAS > 4) patients using generalized linear models (GLMs) with identity link function to control for covariates, including age and sex. Finally, bivariate comparisons were conducted to compare the characteristics of patients who had visited HCPs for their CC and those who had not; the comparisons were conducted by combining patients from South Korea and Taiwan together due to the limited sample sizes. All statistical analyses were performed using IBM SPSS Statistics Version 25 [22] and R version 3.6.3 [23]. *P* values of less than 0.05 were considered statistically significant.

## Results

### Study Subjects

**Fig. 1:**
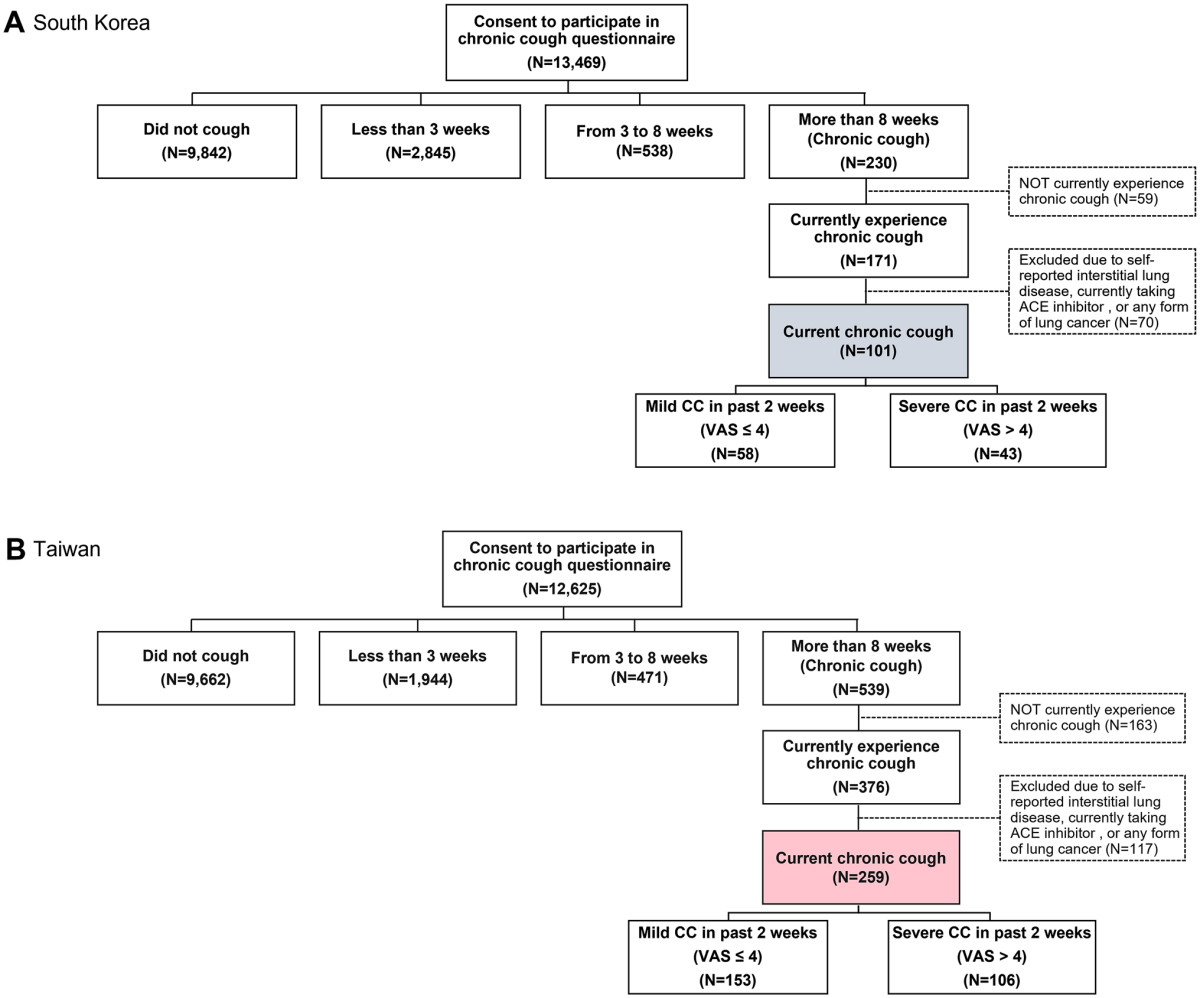
Respondent flowchart

Based on the selection criteria, a total of 360 subjects with current CC (101 from South Korea and 259 from Taiwan) were identified (Fig. 1A and B). Patients in South Korea were aged 43.93 ± 13.99 years (mean ± SD) and 55.4% were females and those in Taiwan were aged 48.69 ± 14.35 years and 45.2% were females (Table 1).

**Table 1:** Cough-related clinical characteristics of chronic cough patients in the 2020 South Korea and Taiwan National Health and Wellness Survey

		South Korea (*N* = 101)			Taiwan (*N* = 259)		
		*N*	Mean	SD	*N*	Mean	SD
Age		101	43.93	13.99	259	48.69	14.35
Years experienced CC		71	3.45	5.13	167	5.75	7.28
Cough severity VAS score over the past 2 weeks		101	4.50	2.15	259	4.46	1.92
Cough severity VAS score on the worst day during the past 2 weeks		101	5.67	2.32	259	5.76	2.31
LCQ total score		101	13.85	3.41	259	13.99	3.27
HARQ total score		101	25.59	15.58	259	22.18	13.30
		**%**			**%**		
Sex	Female	55.4%			45.2%		
	Male	44.6%			54.8%		
Cough more severe at a certain time of year	No	32.4%			53.4%		
	Yes	67.6%			46.6%		
Cough more severe in	Spring (March, April, May)	36.0%			11.1%		
	Summer (June, July, August)	0.0%			1.9%		
	Fall/Autumn (September, October, November)	0.0%			11.1%		
	Winter (December, January, February)	64.0%			75.9%		
How often do you cough up phlegm when coughing	Never	6.9%			6.6%		
	Rarely	23.8%			37.8%		
	Sometimes	42.6%			32.4%		
	Often	18.8%			18.1%		
	Always	7.9%			5.0%		
Cough start/exacerbate with cold or flu-like illness	No	50.5%			37.8%		
	Yes	32.7%			38.6%		
	Do not know	16.8%			23.6%		
Smoking status	Not smoked in the last 12 months	56.4%			74.1%		
	Not currently smoking but smoked in last 12 months	3.0%			1.9%		
	Currently smoking	40.6%			23.9%		
Experienced urinary incontinence while coughing	No	59.4%			82.2%		
	Yes	40.6%			17.8%		
Experienced post-nasal drip	No	33.7%			27.4%		
	Yes	66.3%			72.6%		

*CC* chronic cough; *HARQ* Hull Airway Reflux Questionnaire; *LCQ* Leicester Cough Questionnaire; *VAS* visual analogue scale

### CC Patient Characteristics

**Fig. 2:**
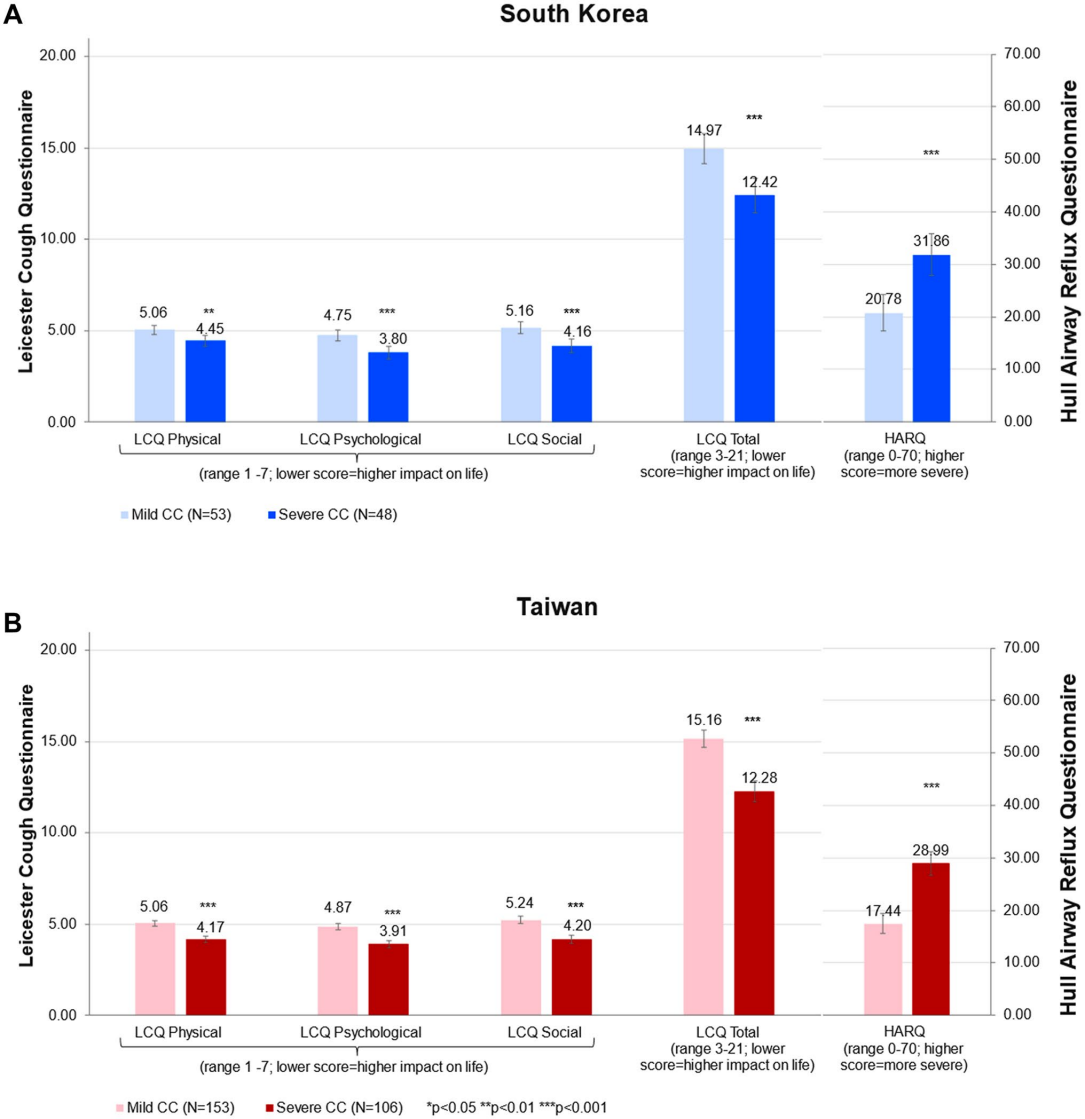
Comparison of LCQ and HARQ scores between mild and severe CC patients in South Korea (upper panel) and Taiwan (lower panel). Note: Data are presented as mean ± standard error (SE). *CC* chronic cough; *HARQ* Hull Airway Reflux Questionnaire; *LCQ* Leicester Cough Questionnaire; *QoL* quality of life. Asterisks (***) indicate significance of *p* < 0.001

Patients with current CC in South Korea had cough persisting for 3.45 ± 5.13 years, while those in Taiwan had cough for 5.75 ± 7.28 years (Table 1). The cough severity VAS scores in South Korea and Taiwan over the past two weeks were 4.50 ± 2.15 and 4.46 ± 1.92, respectively. In South Korea, 42.6% of patients were considered to have severe CC (VAS > 4) and 40.9% in Taiwan had severe CC (Fig. 1). Total LCQ scores in South Korea and Taiwan were 13.85 ± 3.41 and 13.99 ± 3.27, while HARQ scores were 25.59 ± 15.58 and 22.18 ± 13.30, respectively (Table 1). Cough severity was significantly associated with greater cough burden as measured by the LCQ (total and all domain scores) and HARQ scores (Fig. 2). Those with severe CC had mean LCQ total scores of 12.42 and 12.28 in South Korea and Taiwan, respectively, and the differences with those of mild CC were 2.55 and 2.88, respectively, exceeding the minimal important difference of 1.3 for the scale.

More than 40% of patients (South Korea: 67.6%; Taiwan: 46.6%) felt that cough was more severe at a certain time of year; cough was more severe during winter (South Korea: 64.0%; Taiwan: 75.9%) and spring (South Korea: 36.0%); however, spring was not remarkable in Taiwan (11.1%) (Table 1). A quarter of patients (South Korea: 26.7%; Taiwan: 23.1%) often or always coughed up phlegm. In South Korea, 40.6% were still smoking, and 23.6% in Taiwan were currently smoking. The proportion of patients experiencing urinary incontinence when coughing was 40.6% in South Korea and 17.8% in Taiwan, respectively. Urinary incontinence was significantly more frequent in females than in males (41.6 vs. 8.0%; *p* < 0.001).

### CC Patient Journey: Healthcare Visits

Among CC patients in South Korea and Taiwan, 70.3% and 57.9% had, respectively, reported having ever spoken with a physician for cough (Table 2). The most common types of HCPs patients first sought medical consultation were otolaryngologist (South Korea: 33.7%; Taiwan: 45.9%), primary care physician (20.8%; 11.2%), and pulmonologist (11.9%; 9.3%) (Table 2). Other common HCPs consulted after the patients’ first HCP visit were otolaryngologist, primary care physician, and pulmonologist in South Korea and otolaryngologist, traditional oriental physician, and pulmonologist in Taiwan (Supplementary Table 1). In South Korea, 53.7% of the HCPs first seen were in clinics, followed by semi-hospitals (31.7%) and tertiary hospitals (14.6%). In Taiwan, 68.9% of the HCPs first seen were in clinics, followed by area hospitals (12.3%), regional hospitals (10.4%), and medical centers (8.5%) (Table not shown).

**Table 2:** History and experiences of seeking HCPs for CC in South Korea and Taiwan

		South Korea (*N* = 101)	Taiwan (*N* = 259)
		%	%
History and experiences of seeking HCPs for CC			
Ever spoken with a physician for cough	No	29.7	42.1
	Yes	70.3	57.9
HCP first seen for CC	None	18.8	18.1
	Primary care physician (Family physician; Internist)	20.8	11.2
	Pulmonologist	11.9	9.3
	Allergist	2.0	4.6
	Otolaryngologist	33.7	45.9
	Head and neck surgeon	0.0	0.0%
	Gastroenterologist	11.9	1.5
	Urologist	0.0	0.0
	Traditional oriental Physician	1.0	7.3
	Other	0.0	1.9
How knowledgeable is your physician in how to evaluate and treat CC (*N* = 71/150)	Not at all knowledgeable	0.0	4.7
	A little knowledgeable	16.9	32.0
	Somewhat knowledgeable	54.9	52.0
	Extremely knowledgeable	28.2	11.3
Physicians have a good understanding of how CC impacts your life (*N* = 71/150)	No	23.9	28.7
	Yes	76.1	71.3
Feel that your doctor(s) (*N* = 71/150)	Ordered too many tests (such as x-rays, CT scans, breathing tests)	7.0	4.7
	Did not order enough tests (such as x-rays, CT scans, breathing tests)	16.9	26.0
	Ordered the appropriate tests	59.2	52.0
	None of the above	16.9	17.3
Feel that your doctor(s) (*N* = 71/150)	Sent you to too many additional doctors, like specialists	1.4	3.3
	Did not send you to enough or the right doctors/specialists	15.5	38.7
	Sent you to the right doctors/specialists	49.3	28.7
	None of the above	33.8	29.3
Diagnostics tests conducted to evaluate CC			
Chest imaging (X-ray or CT scan)		40.6	40.2
Spirometry		35.6	22.4
Allergy test		19.8	14.7
Sinus imaging (X-ray or CT scan)		9.9	5.8
GI testing (Endoscopy/Barium swallow, esophageal pH testing)		8.9	12.4
Bronchoscopy		14.9	8.1
I do not know		6.9	12.7
None		25.7	34.7

*CC* chronic cough; *CT* computed tomography; *HCP* healthcare provider

**Fig. 3:**
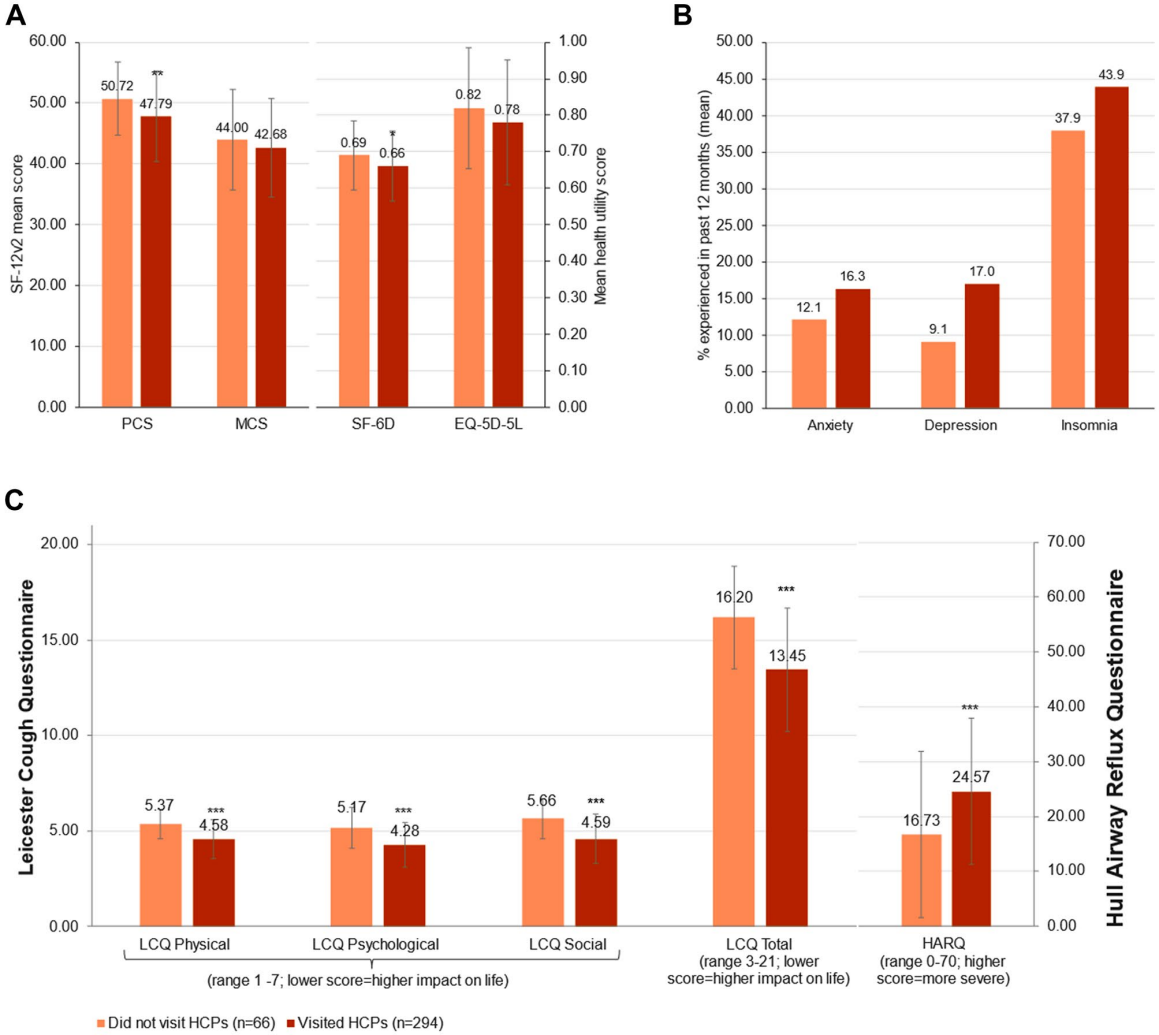
Comparison of general health-related QoL, cough-specific QoL, and HARQ scores between CC patients who had not visited HCPs and those who had visited HCPs. Note: Data are presented as mean ± standard deviation (SD). *CC* chronic cough; *EQ-5D-5L* 5-level EQ-5D version, *HARQ* Hull Airway Reflux Questionnaire; *LCQ* Leicester Cough Questionnaire; *SF-12* 12-item short-form survey; *SF-6D* short-form six dimension; *MCS* mental component summary; *PCS* physical component summary; *QoL* quality of life. Asterisks (**) indicate significance of *p* < 0.01 and (***) indicate significance of *p* < 0.001

Table 3 describes pooled analyses of patients who had not visited any HCPs compared to those who had visited HCPs for CC. Age, cough duration, sex, or smoking status did not significantly differ between two groups. However, patients who had visited HCPs for CC had more severe cough, experienced more cough-related urinary incontinence and post-nasal drip than those who had not. In addition, patients who had visited HCPs reported significantly greater burden and cough symptom severity than those who had not, as measured by poorer PCS score in the SF-12v2 (47.79 ± 7.47 vs. 50.72 ± 6.01; *p* = 0.003), SF-6D score (0.66 ± 0.10 vs. 0.69 ± 0.09; *p* = 0.029), LCQ total score (13.45 ± 2.68 vs. 16.20 ± 3.23; *p* < 0.001) and domain scores, and also higher HARQ scores (24.57 ± 13.38 vs. 16.73 ± 15.16; *p* < 0.001). The proportion of self-reported anxiety, depression, or insomnia was numerically higher among those who had visited HCPs for CC, but the difference was not statistically different (Fig. 3).

**Table 3:** Comparison of cough characteristics between patients who did not visit HCPs (*n* = 66) vs. CC patients who had visited HCPs (*n* = 294) for CC

		CC patients who did not visit HCPs (*n* = 66)		CC patients who visited HCPs (*n* = 294)		*p* value
		Mean	SD	Mean	SD	
Age		47.09	13.95	47.42	14.51	0.868
Years experienced CC (*n* = 32/206)		5.28	6.98	5.03	6.77	0.849
Cough severity VAS score over the past 2 weeks		3.89	1.71	4.6	2.02	0.009
Cough severity VAS score on the worst day during the past 2 weeks		4.83	2.12	5.94	2.3	< 0.001
		%		%		
Sex	Female	40.9%		49.7%		0.198
	Male	51.9%		50.3%		
How often do you cough up phlegm when coughing	Never	13.6%		5.1%		0.033
	Rarely	28.8%		35.0%		
	Sometimes	42.4%		33.7%		
	Often	12.1%		19.7%		
	Always	3.0%		6.5%		
Smoking status	Not smoked in the last 12 months	54.5%		72.4%		0.17
	Not currently smoking but smoked in last 12 months	3.0%		2.0%		
	Currently smoking	32.4%		25.5%		
Experienced urinary incontinence while coughing	No	86.4%		73.5%		0.027
	Yes	13.6%		26.5%		
Experienced post-nasal drip	No	53.0%		23.8%		< 0.001
	Yes	47.0%		76.2%		

*CC* chronic cough; *HCP* healthcare provider; *VAS* visual analogue scale

### CC Patient Journey: Diagnoses and Treatments

Among those who had spoken with a physician for CC, about one-quarter of them felt that their physician was not very knowledgeable or did not have good understanding of how CC impacts their life (Table 2). Although more than half patients in South Korea and Taiwan (59.2%; 52.0%) felt their doctor(s) ordered the appropriate number of tests, 38.7% and 15.5% of Taiwanese and Korean patients, respectively, felt their doctor(s) did not send them to enough or the right doctors/specialists. The most common tests conducted were chest imaging (X-ray or CT scan) (South Korea: 40.6%; Taiwan: 40.2%), spirometry (35.6%; 22.4%), and allergy test (19.8%; 14.7%), while about 30% of the patients had not taken any tests. A proportion of the patients were not aware of the types of tests (South Korea: 6.9%; Taiwan: 12.7%) (Table 2).

Around one-fifth of patients had not received a diagnosis (“none” or “do not know”) for their CC from a physician (Table 4). The most common diagnoses were allergic rhinitis (36.6%), chronic rhinitis (29.7%), and habit cough (24.8%) in South Korea and were post-nasal drip (38.6%) and rhinitis (allergic: 32.8%; nasal: 30.5%) in Taiwan.

**Table 4:** Physician-diagnosed underlying conditions or behaviors related to CC

	South Korea (*N* = 101)	Taiwan (*N* = 259)
Underlying conditions^a^	%	%
Allergic rhinitis	36.6	32.8
Asthma	20.8	12.7
Chronic bronchitis	22.8	21.6
Chronic obstructive pulmonary disease (COPD)	2.0	2.3
Chronic rhinitis	29.7	7.7
Chronic sinusitis	12.9	10.4
Emphysema	0.0	1.2
Gastro-esophageal reflux disease (GERD)	18.8	22.4
I cough out of habit	24.8	15.1
Nasal allergies	22.8	30.5
Nasal polyps	1.0	4.6
Post-nasal drip	16.8	38.6
Sinus bronchial syndrome	2.0	3.9
Vocal cord dysfunction	3.0	3.5
None	9.9	9.3
Others	3.0	3.9
I do not know	11.9	9.7

*CC* chronic cough

^a^Conditions or behaviors related to CC were defined by patients’ responses to the question “Has a doctor ever told you that any of the following conditions or behaviors are related to your chronic cough?” Patients were allowed to choose multiple underlying diseases. If there were no underlying diseases, patients were allowed to choose only “I don’t know” or “None”

Most patients reported ever taking medication(s) for CC (South Korea: 85.1%; Taiwan: 80.7%) (Table 5). Commonly used medications were anti-tussives (60.4%), antibiotics (18.8%), and 1st-generation antihistamines (17.8%) in South Korea, while anti-tussives (50.2%), nasal steroids (25.9%), and cough drops (25.5%) were common in Taiwan (Table 4). Use of codeine or hydrocodone-containing products was reported by 11.9% and 28.2% of patients in South Korea and Taiwan, respectively, and the duration of regular use was 9.25 ± 8.11 months in South Korea and 18.68 ± 45.65 months in Taiwan. However, more than half of patients perceived anti-tussive medications (including anti-tussives, anti-epileptics, 1st generation antihistamines, or cough drops) as “not at all” or “a little bit” useful to treat their CC (Table 5).

**Table 5:** Self-reported history of medications given for treating CC

South Korea					
	Medication Usage	How well did the medication treat your chronic cough? (%)			
Medication	(*N* = 101)	Not at all	A little bit	Somewhat	A great deal
None	14.9%	–	–	–	–
Anti-tussives (such as common cold medicine)	60.4%	11.5%	45.9%	34.4%	8.2%
Anti-epileptics (such as gabapentin)	2.0%	0.0%	100.0%	0.0%	0.0%
Proton pump inhibitors	14.9%	0.0%	46.7%	46.7%	6.7%
H2 blockers	1.0%	100.0%	0.0%	0.0%	0.0%
Nasal Steroids	5.9%	16.7%	33.3%	50.0%	0.0%
Inhaled steroids	2.0%	0.0%	0.0%	50.0%	50.0%
Oral steroids	2.0%	0.0%	50.0%	0.0%	50.0%
Beta-agonists	3.0%	0.0%	66.7%	33.3%	0.0%
1st-generation antihistamines	17.8%	5.6%	50.0%	38.9%	5.6%
2nd-generation antihistamines	13.9%	0.0%	35.7%	50.0%	14.3%
Cough drops	16.8%	23.5%	58.8%	11.8%	5.9%
Antibiotics	18.8%	21.1%	31.6%	31.6%	15.8%
ICS-LABA	6.9%	0.0%	42.9%	42.9%	14.3%
Prescribed Traditional Oriental Medicine	10.9%	0.0%	45.5%	54.5%	0.0%
Traditional Oriental Medicine bought over the counter	5.9%	33.3%	16.7%	50.0%	0.0%
Others	4.0%	50.0%	50.0%	0.0%	0.0%
Ever taken codeine or hydrocodone-containing products for ≥ 1 month	11.9%	Duration of regular use (months)			
		Mean		SD	
		9.25		8.11	

Taiwan					
	Medication usage	How well did the medication treat your chronic cough? (%)			
Medication	(*N* = 259)	Not at all	A little bit	Somewhat	A great deal
None	19.3%	–	–	–	–
Anti-tussives	50.2%	16.9%	60.8%	20.8%	1.5%
Anti-epileptics	1.9%	40.0%	40.0%	20.0%	0.0%
Proton pump inhibitors	11.2%	10.3%	51.7%	31.0%	6.9%
H2 blockers	5.8%	13.3%	53.3%	26.7%	6.7%
Nasal Steroids	25.9%	10.4%	64.2%	20.9%	4.5%
Inhaled steroids	9.7%	12.0%	44.0%	36.0%	8.0%
Oral steroids	5.8%	0.0%	60.0%	20.0%	20.0%
Beta-agonists	13.5%	11.4%	42.9%	34.3%	11.4%
1st-generation antihistamines	13.1%	5.9%	64.7%	29.4%	0.0%
2nd-generation antihistamines	11.6%	13.3%	50.0%	33.3%	3.3%
Cough drops	25.5%	25.8%	66.7%	7.6%	0.0%
Antibiotics	12.4%	15.6%	50.0%	28.1%	6.3%
ICS-LABA	8.9%	4.3%	39.1%	47.8%	8.7%
Prescribed Traditional Oriental Medicine	21.6%	16.1%	58.9%	19.6%	5.4%
Traditional Oriental Medicine bought over the counter	22.8%	23.7%	57.6%	16.9%	1.7%
Others	2.3%	0.0%	50.0%	50.0%	0.0%
Ever taken codeine or hydrocodone-containing products for ≥ 1 month	28.2%	Duration of regular use (months)			
		Mean		SD	
		18.68		45.65	

*ICS-LABA* inhaled corticosteroid and long-acting β2-agonist

## Discussion

The present study investigated cough characteristics and healthcare journey of CC patients in community-based populations of South Korea and Taiwan. As reflected by cough PRO scores, the severity and impact of cough was considerable even among CC patients in the community. The LCQ and HARQ scores in this study had exceeded the normative values of healthy individuals suggested from other countries [24, 25]. We examined their healthcare journeys and found several unmet needs in physician’s knowledge and understanding of CC, diagnostic investigations, and treatment effectiveness. The observed patterns were similar between two countries, despite their differences in demographics, cough seasonality, and healthcare system. To our knowledge, this is the first description of community-based CC patients’ cough characteristics and healthcare journeys in South Korea and Taiwan and is also one of the very few reported globally [26–28].

We speculate that the impact of cough, or cough severity perceived by a patient, is a key factor defining CC as a disease and that cough-related healthcare utilization is a proxy marker of the impact that patients experienced with CC. In the present study, about one-fifth of patients in South Korea and Taiwan did not visit any HCPs for their CC; and HCP visits were significantly associated with several cough characteristics, e.g., cough severity, cough-specific QoL, cough-induced urinary incontinence, or post-nasal drip sensation, but not with cough duration. These findings cannot make any firm conclusions because other unmeasured factors may underlie HCP visits, e.g., cough frequency, other complications, or socioeconomic status; however, they suggest that cough severity and impact are likely important characteristics that define CC as a disease.

In CC patients’ health journey, two issues were noted: (1) lack of effective anti-tussive and (2) proper diagnosis. More than 50% of patients had taken anti-tussives (South Korea: 60.4%; Taiwan: 50.2%), and the mean duration of regularly taking codeine was around one year, but more than half of them found very little effectiveness. These findings are consistent with recent studies of European and Korean patients that reported limited effectiveness of currently available anti-tussives [11, 29]. Indeed, these remain as major unmet needs for refractory or unexplained CC patients.

About 20% of patients reported not receiving any diagnosis for CC. Furthermore, 20–30% of patients felt that their physicians were not as knowledgeable in CC management, nor have a good understanding of the impact. Moreover, chest imaging, which is routinely recommended in cough guidelines, was performed in about 40% of the patients, which is surprising given that bronchoscopy (8.1–14.9%) and GI testing (8.9–12.4%) were frequently done. These findings imply that there are unmet needs to provide optimal/adequate managements for CC in the community and that further efforts are warranted to implement international or national cough guidelines to non-specialist clinics.

Interestingly, unlike in South Korea, a male predominance (54.8%) was noted in CC patients in Taiwan, which is similar to those observed at specialist cough clinics in Guangzhou, China [30]. This might be attributed to ethnic or environmental factors distinct to Taiwan and Southeast China [31]. However, the present study was not designed to investigate the sex difference, and the findings warrant further investigation.

Other differences were noted in the patient characteristics between South Korea and Taiwan. Urinary incontinence was more frequent in South Korean patients than in Taiwanese (40.6% vs. 17.8%), although cough scores were comparable. This is likely attributed to the sex difference as urinary incontinence is a common cough-related complication among female patients [32, 33]. In addition, 40.6% of current CC patients in South Korea were current smokers, similar to the Korean National Health and Nutrition Examination Surveys results (43.0–47.7%) [15, 34]. The proportion of current smokers in Taiwan in this study was less than that reported in a previous study (23.6 vs 31.6%) [35], potentially attributed by a decline in smoking prevalence in Taiwan [36, 37].

We observed that current smokers were frequent among CC subjects in both community populations, which contrasted with the observations at specialist cough clinics where most are never or non-current smokers [31, 38]. This might suggest a difference in CC patient characteristics between community and specialist cough clinics. However, it may be partly due to the limitation of current definition used to identify CC, because the duration-based definition do not well differentiate protective cough responses against irritant exposure (such as cigarette smoke) from hypersensitivity cough [39]. In our view, the duration-based simple definition does not capture the key nature of the disease.

There are limitations of this study. The survey is a cross-sectional study; hence no causal relationships could be concluded. All data were self-reported, therefore recall bias and self-representation bias should be acknowledged. Although the NHWS is broadly representative of the Korean and Taiwanese general adult population, individuals without internet access or those unfamiliar with online administration including those of older age or with severe comorbidities and disabilities are likely to be under-represented in this study. This might underlie a younger age distribution of the present study respondents, compared to those attending specialist cough clinics [38, 40]. Secondly, there may be unmeasured demographic or clinical factors that could influence the patients’ health journey and warrant further investigation. Nevertheless, the present survey provided an opportunity to identify cough-related characteristics as well as the patient journey in the community of South Korea and Taiwan. Respondents of the NHWS were recruited using a stratified random sampling procedure, with strata by sex and age, to ensure that the demographic composition is representative of the respective adult population in each country/territory.

In conclusion, this is the first study describing cough characteristics and healthcare utilization of CC patients in the community-based populations of South Korea and Taiwan. Cough is frequently severe and persistent among community-based CC patients. They experience several issues in their health journey, including treatment ineffectiveness and physician’s understanding. Further efforts are warranted to reduce the burden of CC in the community.

## Supplementary Information

Below is the link to the electronic supplementary material.

Supplementary file1 (DOCX 42 KB)
