# Supplementary material for: Cough Characteristics and Healthcare Journeys of Chronic Cough Patients in Community-Based Populations in South Korea and Taiwan
Source: Lung. 2022 Nov 3;200(6):725–36. doi: 10.1007/s00408-022-00586-3 (PMC9675671; doi:10.1007/s00408-022-00586-3)
Supplement: Supplementary file 1 — Supplementary file1 (DOCX 42 KB) [file 408_2022_586_MOESM1_ESM.docx]

**Supplementary Information**

**Title: Cough characteristics and healthcare journeys of chronic cough patients in community-based populations in South Korea and Taiwan**

**Short title:** Chronic cough patient journey in Korea and Taiwan

**Authors:** Woo-Jung Song^1¶^, Chong-Jen Yu^2¶^, Suk Hyun Kang^3^

^¶^These authors contributed equally

**Affiliations:**

1. Department of Allergy and Clinical Immunology, Asan Medical Center, University of Ulsan College of Medicine, Seoul, Korea; *Email:* [*swj0126@amc.seoul.kr*](mailto:swj0126@amc.seoul.kr)

2. Department of Internal Medicine, and Graduate Institute of Clinical Medicine, National Taiwan University Hospital, Hsin-Chu Branch and National Taiwan University College of Medicine; *Email:* [*jefferycjyu@ntu.edu.tw*](mailto:jefferycjyu@ntu.edu.tw)

3. Oncology Market Access, MSD, Seoul, Korea; *Email:* [*sukhyun.kang@merck.com*](mailto:sukhyun.kang@merck.com)

# Methods

*Parameters collected from NHWS*

Demographic measures including age, sex, employment status, household income, marital status, and level of education. Baseline characteristics included body mass index (BMI), smoking status, alcohol use, exercise behaviour and Charlson Comorbidity Index (CCI). CCI weights the presence and seriousness of chronic comorbid diseases. An adapted version of the CCI was created excluding chronic pulmonary disease from the index score because CC is often a symptom of another pulmonary diseases that should not be counted again to measure the comorbid burden [1]. Higher index indicates greater comorbid burden [2–4].

Health-related quality of life (HRQoL) was assessed by 12-Item Short Form Survey Instrument (SF-12v2) which reports two summary scores: physical component summary (PCS) and mental component summary (MCS) scores [5]. Health status utilities were generated from SF-12v2 through the application of the SF-6D algorithm which takes 6 domains from the SF-12v2; SF-6D is a single index measure for health status using general population values [5]. The EQ-5D-5L instrument is a standardized measure of generic HRQoL [6]. Psychological comorbidities measured in this study included self-reported experience of anxiety, depression and insomnia in the past 12 months.

*Outcomes and covariates collected from CC survey*

Cough-specific QoL was measured by Leicester Cough Questionnaire (LCQ) [7], which is one of the most widely used cough-specific HRQoL questionnaires. LCQ is a 19-item, self-administered questionnaire that comprises three main health domains/subscales related to HRQoL measures of CC: physical, psychological, and social. All items are scored on a 7-point Likert response scale (such as, 1 = “all of the time” to 7 = “none of the time”). The LCQ is well-validated and has good internal reliability, repeatability, and responsiveness [7]. The minimal important difference for the LCQ is a total score of 1.3, and the total score has a range of 3-21 points [8]. The minimal important difference for each subscale was: 0.20 for physical, 0.20 for social, and 0.80 for psychological [8]. Each of the three subscales has a range between 1-7 points.

The Hull Airway Reflux Questionnaire (HARQ) is a 14-item, self-administered instrument that measures specific symptoms related to cough. All items are scored on a 0-5 scale (0 = “no problems” to 5 = “severe/frequent problems”), with the total score ranging from 0-70. An average score is 4 out of 70 among people without CC, and the upper limit of normal is 13 [9,10].

# References:

1. Morice AH, Members C. The diagnosis and management of chronic cough. Eur Respir J. 2004 Sep 1;24(3):481–92.

2. Charlson ME, Pompei P, Ales KL, MacKenzie CR. A new method of classifying prognostic comorbidity in longitudinal studies: development and validation. J Chronic Dis. 1987;40(5):373–83.

3. Charlson ME, Charlson RE, Peterson JC, Marinopoulos SS, Briggs WM, Hollenberg JP. The Charlson comorbidity index is adapted to predict costs of chronic disease in primary care patients. J Clin Epidemiol. 2008 Dec;61(12):1234–40.

4. Quan H, Li B, Couris CM, Fushimi K, Graham P, Hider P, et al. Updating and validating the Charlson comorbidity index and score for risk adjustment in hospital discharge abstracts using data from 6 countries. Am J Epidemiol. 2011 Mar 15;173(6):676–82.

5. Brazier JE, Roberts J. The estimation of a preference-based measure of health from the SF-12. Med Care. 2004 Sep;42(9):851–9.

6. EuroQol Group. EuroQol - a new facility for the measurement of health-related quality of life. Health Policy. 1990 Dec 1;16(3):199–208.

7. Birring SS, Prudon B, Carr AJ, Singh SJ, Morgan MDL, Pavord ID. Development of a symptom specific health status measure for patients with chronic cough: Leicester Cough Questionnaire (LCQ). Thorax. 2003 Apr 1;58(4):339–43.

8. Raj AA, Pavord DI, Birring SS. Clinical Cough IV:What is the Minimal Important Difference for the Leicester Cough Questionnaire? In: Chung KF, Widdicombe J, editors. Pharmacology and Therapeutics of Cough [Internet]. Berlin, Heidelberg: Springer; 2009 [cited 2020 Apr 1]. p. 311–20. (Handbook of Experimental Pharmacology). Available from: https://doi.org/10.1007/978-3-540-79842-2_16

9. Hull Cough Hypersensitivity Questionnaire [Internet]. International Society for the Study of Cough. [cited 2020 Apr 27]. Available from: http://www.issc.info/HullCoughHypersensitivityQuestionnaire.html

10. Morice AH, Faruqi S, Wright CE, Thompson R, Bland JM. Cough hypersensitivity syndrome: a distinct clinical entity. Lung. 2011 Feb;189(1):73–9.

**Supplementary Table 1. Other HCPs (other than the first HCP) seen by study participants to evaluate CC**

|  | | **South Korea (N=82)** | **Taiwan (N=212)** |
| --- | --- | --- | --- |
|  |  | **%** | **%** |
| None | *No* | 98.8% | 80.2% |
|  | *Yes* | 1.2% | 19.8% |
| Primary care physician (Family physician; Internist) | *No* | 70.7% | 82.5% |
|  | *Yes* | 29.3% | 17.5% |
| Pulmonologist | *No* | 78.0% | 81.6% |
|  | *Yes* | 22.0% | 18.4% |
| Allergist | *No* | 89.0% | 90.1% |
|  | *Yes* | 11.0% | 9.9% |
| Otolaryngologist | *No* | 53.7% | 56.6% |
|  | *Yes* | 46.3% | 43.4% |
| Head and neck surgeon | *No* | 100.0% | 99.1% |
|  | *Yes* | 0.0% | 0.9% |
| Gastroenterologist | *No* | 84.1% | 91.0% |
|  | *Yes* | 15.9% | 9.0% |
| Urologist | *No* | 100.0% | 100.0% |
|  | *Yes* | 0.0% | 0.0% |
| Traditional Oriental Physician | *No* | 89.0% | 80.2% |
|  | *Yes* | 11.0% | 19.8% |
| Other | *No* | 100.0% | 98.1% |
|  | *Yes* | 0.0% | 1.9% |
